# Studies on Synthesis and Structure-Activity Relationship (SAR) of Derivatives of a New Natural Product from Marine Fungi as Inhibitors of Influenza Virus Neuraminidase

**DOI:** 10.3390/md9101887

**Published:** 2011-10-11

**Authors:** Jing Li, Dingmei Zhang, Xun Zhu, Zhenjian He, Shu Liu, Mengfeng Li, Jiyan Pang, Yongcheng Lin

**Affiliations:** 1 School of Chemistry and Chemical Engineering, Sun Yat-Sen University, Guangzhou 510275, China; E-Mail: zsulijing@163.com; 2 Guangdong Province Key Laboratory of Functional Molecules in Oceanic Microorganism, Bureau of Education, Sun Yat-sen University, Guangzhou 510080, China; E-Mails: dingmeizhang@yahoo.com.cn (D.Z.); zhuxun8@mail.sysu.edu.cn (X.Z.); hzj100@163.com (Z.H.); 3 Key Laboratory of Tropical Disease Control, Ministry of Education, Sun Yat-sen University, 74 Zhongshan Road II, Guangzhou 510080, China; 4 Department of Microbiology, Zhongshan School of Medicine, Sun Yat-sen University, 74 Zhongshan Road II, Guangzhou 510080, China; 5 Department of Anatomy, Zhongshan School of Medicine, Sun Yat-sen University, 74 Zhongshan Road II, Guangzhou 510080, China; E-Mail: liushu@mail.sysu.edu.cn

**Keywords:** aromatic ether, marine fungus, neuraminidase inhibitor

## Abstract

Based on the natural isoprenyl phenyl ether from a mangrove-derived fungus, 32 analogues were synthesized and evaluated for inhibitory activity against influenza H1N1 neuraminidase. Compound **15** (3-(allyloxy)-4-hydroxybenzaldehyde) exhibited the most potent inhibitory activity, with IC_50_ values of 26.96 μM for A/GuangdongSB/01/2009 (H1N1), 27.73 μM for A/Guangdong/03/2009 (H1N1), and 25.13 μM for A/Guangdong/ 05/2009 (H1N1), respectively, which is stronger than the benzoic acid derivatives (~mM level). These are a new kind of non-nitrogenous aromatic ether Neuraminidase (NA) inhibitors. Their structures are simple and the synthesis routes are not complex. The structure-activity relationship (SAR) analysis revealed that the aryl aldehyde and unsubstituted hydroxyl were important to NA inhibitory activities. Molecular docking studies were carried out to explain the SAR of the compounds, and provided valuable information for further structure modification.

## 1. Introduction

**Chart 1:**
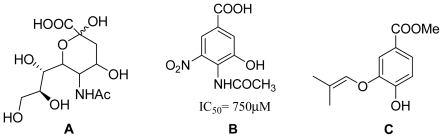


Influenza virus is a major global threat that can result in periodic epidemics [1], causing high morbidity and mortality [2] as flu infections. Neuraminidase (NA) is a surface glycoprotein of influenza virus that catalyzes the cleavage of terminal sialic acid (Chart 1A) residues to facilitate the release as well as spread and transmission of the progeny virus particles from host to host. A number of classes of NA inhibitors have been developed in the past few years, which have shown to be effective in controlling influenza infection, however, most of them are stereochemically complex or orally ineffective, and their synthesis routes are lengthy. In an attempt to develop chemically simple inhibitors, benzoic acid derivatives (Chart 1B) have been studied as important NA inhibitors [3–6]. Their aromatic benzene ring has been used to replace the pyranose of sialic acid in designing NA inhibitors, however, this class of inhibitors have high affinity for influenza virus but high IC_50_ values (~mM level) [7]. Hence, further search for novel NA inhibitors, with simple chemistry and strong potency, has attracted extensive interest.

**Chart 2:**
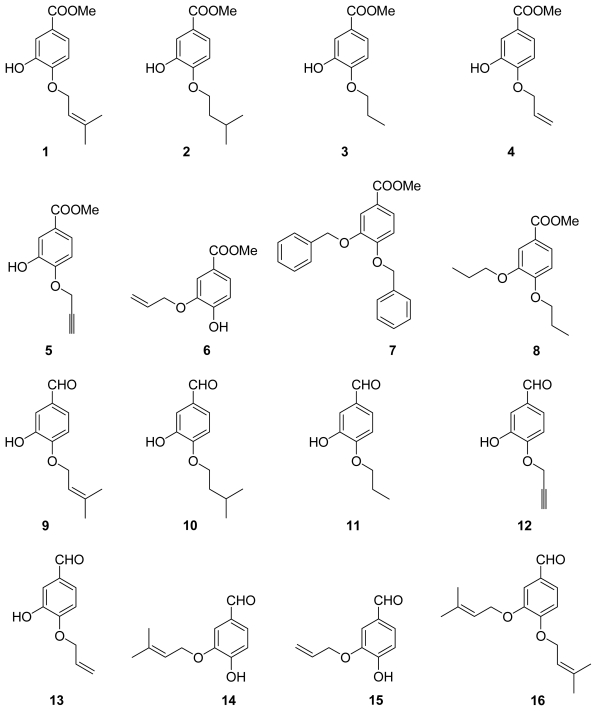

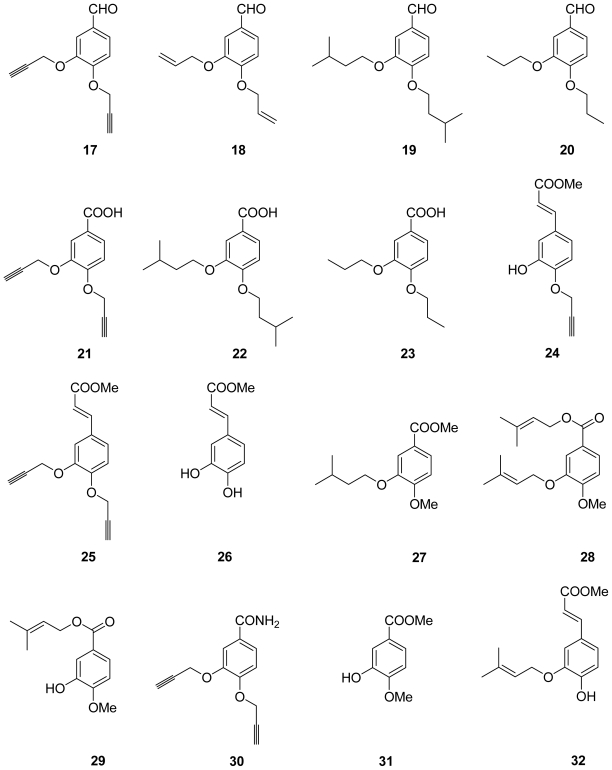
Structures of derivatives.

The metabolites of marine fungi have yielded a lot of novel and bioactive secondary metabolites. Previously we reported the new isoprenyl phenyl ether (Chart 1C) from a mangrove fungus (NO. B60) from the South China Sea with slight cytotoxicity on hepG2 cell line [8], which is similar to benzoic acid NA inhibitors in structure. Therefore, we have keenly become interested in designing and synthesizing novel NA inhibitors by using the isoprenyl phenyl ether as model compound. In this paper, we described the synthesis, biological activities and structure-activities relationship (SAR) analysis of aromatic ether analogues, **1**–**32** (Chart 2). The modification of compounds is focused on: carboxylic acid, ester, aldehyde or amide substituted at 1-position of the aromatic ether, and a variety of substituents at 3- or 4-hydroxyl group of aromatic ether. Compared to the benzoic acid derivatives, our compounds, which do not have the N atom in the molecule, have much more simple structures and better activities. They are also easily obtained with good yields from commercially available materials. In addition, Wang *et al.* [9] reported the active site of NA: a negatively charged group is favored for binding to the pocket formed by Arg118, Arg292 and Arg371, and a positively charged group is needed in the Asp151 and Arg156 pocket. There are two hydrophobic regions in which hydrophobic groups can be introduced to enhance binding intensities: one is formed by Trp178, Ile222 and Arg224, and the other is created by rotating Glu276. We carried out a surflex-docking study of compounds to further account for the experimental results by comparing the binding action of these inhibitors to the active sites of NA.

## 2. Results and Discussion

### 2.1. Chemistry

**Scheme 1:**
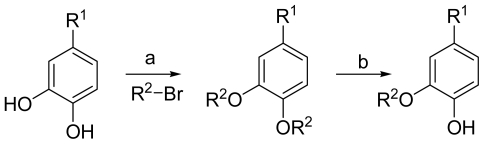
(**a**) K_2_CO_3_/Acetone; (**b**) Pd/C.

The general synthetic route of **1**–**32** is outlined in Scheme 1. We chose 3,4-dihydroxybenzoic acid methyl ester, 3,4-dihydroxybenzaldehyde, 3,4-dihydroxybenzoic acid, isovanillic acid, and caffeic acid as the starting materials. A series of aromatic ethers were synthesized through the Williamson ether synthesis, followed by selective mono-deallylation using 10% Pd/C or aminationa of the intermediate to provide part of target compounds [10]. Most of the compounds were easily obtained with excellent yields (60–95%). Compound **2**, **3**, **5**, **8**, **14**, **19**, **24**, **25**, **27**–**30** and **32** were new derivatives. All new compounds were fully characterized by HRMS and NMR. The purities (>95%) of all target compounds were checked by HPLC using a LC-2010c equipped with UV detector.

### 2.2. H1N1 Virus Neuraminidase Inhibition

**Figure 1:**
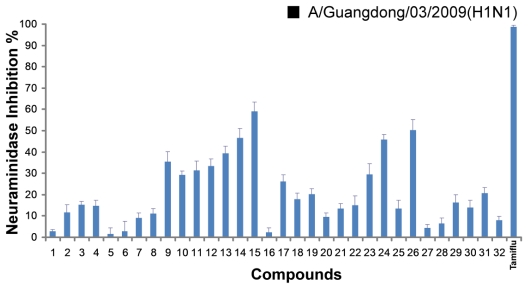
The inhibitory effect of 32 compounds on the NA activity of the pandemic influenza H1N1 2009 viruses. Data points are presented as means ± SD of triplicated experiments.

All compounds were evaluated for inhibitory activities on the pandemic influenza H1N1 virus [(A/Guangdong/03/2009 (H1N1)] neuraminidase *in vitro*, the NA inhibitors oseltamivir (Tamiflu) was used as positive control. The results (Figure 1) showed that most of the derivatives inhibited H1N1 virus neuraminidase in the range of 15–59% inhibition at the 50 μM concentration. Among them, compound **15** and **26** exhibited 50% or higher activities. In this screen, the model compound (Chart 1C) had no activity. The SAR analysis revealed that: (1) Compared with compounds **16**–**18**, **20** and **25**, compounds **9**, **11**–**13**, and **24** exhibited higher activities, it meant that substitution of 3- and 4-OH would reduce the activities; (2) compound **13** showed weaker activity than compound **15**, and it indicated that OH group at C-4 position could improve the activity; (3) for compounds **9**–**13**, it seems that allyl ether is better than isopentyl ether, propyl ether, propargyl ether and isopentene ether, which indicated that maybe the smaller group substituted in this series of compounds is helpful for the activities; (4) previous studies showed that carboxylic acid is an important group in maintaining activities. However, acid changed to aldehyde in 1-position of aromatic compounds was more favorable for the bioactivities in compounds **17**, **21**, **25** and **30** in our present study.

**Figure 2:**
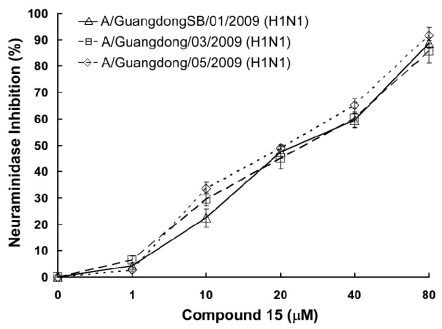
Compound **15** inhibits NA enzymatic activity. Compound **15** inhibits enzymatic activity of NA derived from A/GuangdongSB/01/2009 (H1N1), A/Guangdong/03/2009 (H1N1) and A/Guangdong/05/2009 (H1N1) viruses. Viruses were incubated with increasing concentrations of compound **15** and NA enzymatic activity was determined by a chemiluminescence assay. Results were presented as means ± SD of triplicated experiments.

The effect of compound **15** was further investigated on the neuraminidase activity of three pandemic influenza H1N1 2009 virus strains [A/GuangdongSB/01/2009 (H1N1), A/Guangdong/03/2009 (H1N1) and A/Guangdong/05/2009 (H1N1)]. The results (Figure 2) revealed significant inhibitory effects of compound **15** on all three tested viral NA, with IC_50_ values of 26.96 μM for A/GuangdongSB/01/2009 (H1N1), 27.73 μM for A/Guangdong/03/2009 (H1N1), and 25.13 μM for A/Guangdong/05/2009 (H1N1), respectively. At the same time, compound **15** showed low cytotoxity (>600 μM) to virus host cell—Madin-Darby canine kidney (MDCK) cells—and showed good selectivity index.

### 2.3. Molecular Docking Study

Molecular docking was performed to further assess the SAR of different substituents of the inhibitors by their interactions with the NA residues. The studies focused on the ether chains, the position of OH and the groups at C-1 position.

**Figure 3:**
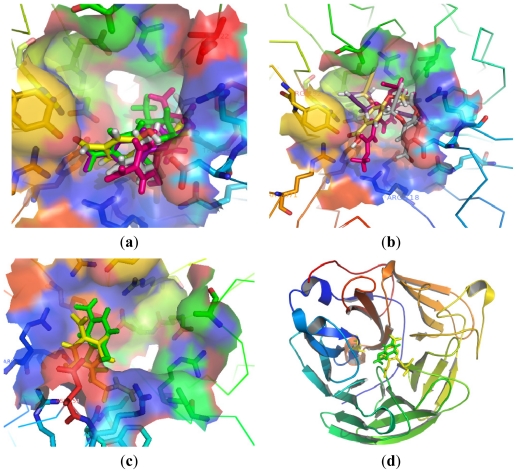

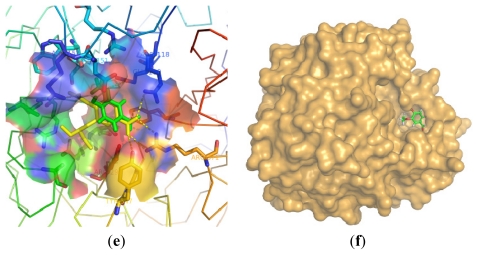
(**a**) Comparison of the positions of compounds **9** (pink), **10** (green), **11** (purple), **12** (yellow) and **13** (gray) in the NA; (**b**) Comparison of the positions of **17** (gray), **21** (yellow), **25** (pink) and **30** (purple); (**c**) Comparison of the positions of **13** (yellow) and **15** (green); (**d**) Comparison of compound **15** (green) and oseltamivir (yellow) in neuraminidase; (**e**) The interaction between the Arg residues and compound **15** (green) in comparison with oseltamivir (yellow); (**f**) Molecular surfaces of neuraminidase with bound compound **15**.

Compounds **9**–**13** with different ether chains in C-4 position produced little difference in activity. Introducing the hydrophobic groups in the small hydrophobic pocket formed by Trp178 and Ile222 as well as the side chain of Arg224 could enhance binding action. In Figure 3(a), all of the ether chains were near the pocket, but the allyl ether chain terminal displayed the best activity compared with other substituents. The triarginyl cluster of Arg118, Arg292, and Arg371 at the pocket is the predominant factor for orienting and stabilizing inhibitors. Compounds **17**, **21**, **25**, **30** had different groups in C-4 position. Relative to **21**, **25** and **30**, compound **17** with an aldehyde group which made strong charge–charge interactions with the triarginyl pocket was more favorable for binding action (Figure 3(b)). From Figure 3(c), we found that the OH group of compound **15** was much closer to the nucleophilic oxygen of Asp151 compared to compound **13**. Asp151 also showed significant favorable electrostatic interaction. The flexibility of the molecule during the docking calculation permitted turning the tail of the ether chains, thus the steric hindrance of the structure did not have a strong effect in binding with Arg156. Molecular docking studies further supported the aforementioned SAR analysis.

The most potent compound **15** was docked into the active sites of NA. Oseltamivir is an efficacious and commonly used neuraminidase inhibitor. The binding of compound **15** with NA was shown in Figure 3(d) and Figure 3(e). The activity of compound **15** could be explained by its interactions with the active residues of NA. The aldehyde group made strong charge–charge interactions with Arg118, Arg292, and Arg371. Asp151 exhibited favorable electrostatic interaction with the OH group at C-4 position. At the same time, the ether chains were suitable for the hydrophobic region formed by Trp178 and Ile222, as well as the side chain of Arg224. Compound **15** provided a binding mode with NA for this series of compounds from its geometric and electronic features.

## 3. Experimental Section

### 3.1. Chemistry

All reagents and solvents were of commercial quality. Melting points were determined on an X-4 micromelting point apparatus and were uncorrected. ^1^H and ^13^CNMR data were recorded on a Varian Inova 400 MB NMR spectrometer operating at 400 and 125 MHz for ^1^H and ^13^C, respectively. All chemical shifts are in ppm (δ) with respect to tetramethylsilane (TMS) as internal standard, and coupling constants (*J*) are in Hz. Mass spectra were obtained on DSQ (low resolution mass spectrometer) and MAT95XP (high resolution mass spectrometer) instruments.

### 3.2. Synthetic Methods of Compounds

#### 3.2.1. General Procedure 1: Synthesis of Compounds **1**–**5**, **7**–**13**, **16**–**25**

The starting materials were dissolved in acetone and 5 equiv K_2_CO_3_ were added. The mixture was stirred for 24 h and then poured into water. The aqueous layer was extracted with ethyl acetate. The combined organic extracts were washed with saturated brine, dried over anhydrous MgSO_4_ and removed the solvent under reduced pressure. Purification by column-chromatography on silica gel:

##### methyl 3-hydroxy-4-((3-methylbut-2-en-1-yl)oxy)benzoate (1)

White solid. Mp 82–84 °C; ^1^H NMR (400 MHz, CDCl_3_) δ 7.60–7.58 (m, 1H), 7.58 (d, *J* = 2.9 Hz, 1H), 6.87 (d, *J* = 9.0 Hz, 1H), 5.69 (s, 1H), 5.47 (t, *J* = 6.8 Hz, 1H), 4.63 (d, *J* = 6.8 Hz, 2H), 3.87 (s, 3H), 1.81 (s, 3H), 1.75 (s, 3H). MS: *m/z* 236 (M^+^).

##### methyl 3-hydroxy-4-(isopentyloxy)benzoate (2)

White solid. Mp 68–70 °C; ^1^H NMR (400 MHz, CDCl_3_) δ 7.59 (s, 1H), 7.58 (d, *J* = 0.8 Hz, 1H), 6.86 (d, *J* = 9.0 Hz, 1H), 5.68 (s, 1H), 4.12 (t, *J* = 6.6 Hz, 2H), 0.97 (s, 3H). ^13^C NMR (101 MHz, CDCl_3_) δ 166.97, 149.96, 145.25, 123.36, 122.46, 115.53, 110.38, 67.46, 51.99, 37.94, 24.96, 22.72. MS: *m/z* 238 (M^+^); HRMS calcd for C_13_H_18_O_4_: 238.1200 (M^+^), found: 238.1201.

##### methyl 3-hydroxy-4-propoxybenzoate (3)

White solid. Mp 66–68 °C; ^1^H NMR (400 MHz, CDCl3) δ 7.60–7.59 (m, 1H), 7.58–7.57 (m, 1H), 6.84 (d, *J =* 9.0 Hz, 1H), 5.73 (s, 1H), 4.05 (t, *J =* 6.6 Hz, 2H), 3.87 (s, 3H), 1.92–1.79 (m, 2H), 1.05 (t, *J =* 7.4 Hz, 3H). ^13^C NMR (101 MHz, CDCl3) δ 166.91, 149.92, 145.49, 123.30, 122.81, 115.68, 110.70, 70.66, 51.78, 22.55, 10.51. MS: *m/z* 210 (M^+^); HRMS calcd for C_11_H_14_O_4_: 210.0887 (M^+^), found: 210.0886.

##### methyl 4-(allyloxy)-3-hydroxybenzoate (4)

White solid. Mp 65–67 °C; ^1^H NMR (400 MHz, CDCl_3_) δ 7.61–7.59 (m, 1H), 7.58 (d, *J* = 2.1 Hz, 1H), 6.87 (d, *J* = 8.3 Hz, 1H), 6.06 (dt, *J* = 16.0, 5.5 Hz, 1H), 5.67 (s, 1H), 5.38 (dd, *J* = 26.5, 14.5 Hz, 2H), 4.67 (d, *J* = 5.5 Hz, 2H), 3.88 (s, 3H). MS: *m/z* 208 (M^+^).

##### methyl 3-hydroxy-4-(prop-2-yn-1-yloxy)benzoate (5)

White solid. Mp 107–109 °C; ^1^H NMR (400 MHz, CDCl_3_) δ 7.61 (s, 1H), 7.59 (d, *J* = 2.1 Hz, 1H), 6.99 (d, *J* = 8.6 Hz, 1H), 5.78 (s, 1H), 4.81 (s, 2H), 3.87 (s, 3H), 2.58 (s, 1H). ^13^C NMR (101 MHz, CDCl_3_) δ 166.79, 148.40, 145.62, 124.49, 122.49, 116.35, 111.73, 57.13, 51.82. MS: *m/z* 206 (M^+^); HRMS calcd for C_11_H_10_O_4_: 206.0574 (M^+^), found: 206.0572.

##### methyl 3,4-bis(benzyloxy)benzoate (7)

Pale yellow solid. Mp 55–57 °C; ^1^H NMR (400 MHz, CDCl_3_) δ 7.67–7.61 (m, 2H), 7.50–7.28 (m, 10H), 6.94 (d, *J* = 8.3 Hz, 1H), 5.20 (d, *J* = 10.5 Hz, 4H), 3.87 (s, 3H). MS: *m/z* 348 (M^+^).

##### methyl 3,4-dipropoxybenzoate (8)

White solid. Mp 39–41 °C; ^1^H NMR (400 MHz, CDCl_3_) δ 7.63 (dd, *J* = 8.4, 2.0 Hz, 1H), 7.54 (d, *J* = 2.0 Hz, 1H), 6.87 (d, *J* = 8.5 Hz, 1H), 4.01 (td, *J* = 6.6, 2.1 Hz, 4H), 3.88 (s, 3H), 1.92–1.78 (m, 4H), 1.05 (t, *J* = 7.4 Hz, 6H). ^13^C NMR (101 MHz, CDCl_3_) δ 167.11, 153.42, 148.67, 123.67, 122.63, 114.71, 112.34, 71.01, 70.68, 51.99, 22.72, 22.64, 10.58, 10.54. MS: *m/z* 252 (M^+^); HRMS calcd for C_14_H_20_O_4_: 252.1356 (M^+^), found: 252.1354.

##### 3-hydroxy-4-((3-methylbut-2-en-1-yl)oxy)benzaldehyde (9)

Brown solid. Mp 65–67 °C; ^1^H NMR (300 MHz, CDCl_3_) δ 9.82 (s, 1H), 7.42 (s, 2H), 6.97 (s, 1H), 5.80 (s, 1H), 5.48 (s, 1H), 4.67 (s, 2H), 1.80 (d, *J* = 15.3 Hz, 6H). MS: *m/z* 206 (M^+^).

##### 3-hydroxy-4-(isopentyloxy)benzaldehyde (10)

Brown oil. ^1^H NMR (300 MHz, CDCl_3_) δ 9.84 (s, 1H), 7.42 (d, *J* = 10.2 Hz, 2H), 6.96 (d, *J* = 8.0 Hz, 1H), 5.72 (s, 1H), 4.18 (t, *J* = 6.4 Hz, 2H), 1.80 (dt, *J* = 21.9, 6.7 Hz, 3H), 1.02 (s, 3H), 1.00 (s, 3H). MS: *m/z* 208 (M^+^).

##### 3-hydroxy-4-propoxybenzaldehyde (11)

White-like solid. Mp 71–73 °C; ^1^H NMR (300 MHz, CDCl_3_) δ 9.83 (s, 1H), 7.43 (dd, *J* = 8.2, 5.0 Hz, 2H), 6.95 (d, *J* = 8.1 Hz, 1H), 5.75 (s, 1H), 4.12 (t, *J* = 6.6 Hz, 2H), 1.91 (dd, *J* = 14.0, 6.6 Hz, 2H), 1.09 (t, *J* = 7.4 Hz, 3H). MS: *m/z* 180 (M^+^).

##### 3-hydroxy-4-(prop-2-yn-1-yloxy)benzaldehyde (12)

Pale white solid. Mp 82–84 °C; ^1^H NMR (300 MHz, CDCl_3_) δ 9.85 (s, 1H), 7.45 (dt, *J* = 8.6, 2.2 Hz, 2H), 7.10 (d, *J* = 8.2 Hz, 1H), 5.77 (s, 1H), 4.87 (d, *J* = 2.4 Hz, 2H), 2.63 (t, *J* = 2.2 Hz, 1H). MS: *m/z* 176 (M^+^).

##### 4-(allyloxy)-3-hydroxybenzaldehyde (13)

Pale white solid. Mp 55–57 °C; ^1^H NMR (400 MHz, CDCl_3_) δ 9.84 (s, 1H), 7.45 (d, *J* = 2.0 Hz, 1H), 7.41 (dd, *J* = 8.3, 2.0 Hz, 1H), 6.97 (d, *J* = 8.3 Hz, 1H), 6.07 (ddd, *J* = 22.8, 10.9, 5.5 Hz, 1H), 5.80 (s, 1H), 5.41 (dddd, *J* = 20.6, 10.5, 2.6, 1.3 Hz, 2H), 4.71 (dt, *J* = 5.5, 1.4 Hz, 2H). MS: *m/z* 178 (M^+^).

##### 3,4-bis((3-methylbut-2-en-1-yl)oxy)benzaldehyde (16)

Colorless oil. ^1^H NMR (300 MHz, CDCl_3_) δ 9.82 (s, 1H), 7.49–7.35 (m, 2H), 6.96 (d, *J* = 8.7 Hz, 1H), 5.56–5.46 (m, 2H), 4.67 (dd, *J* = 13.0, 6.6 Hz, 4H), 1.80 (s, 6H), 1.77 (s, 6H). MS: *m/z* 274 (M^+^).

##### 3,4-bis(prop-2-yn-1-yloxy)benzaldehyde (17)

Pale yellow solid. Mp 100–102 °C; ^1^H NMR (300 MHz, CDCl_3_) δ 9.88 (s, 1H), 7.63–7.44 (m, 2H), 7.18 (d, *J* = 8.2 Hz, 1H), 4.85 (dd, *J* = 9.3, 2.4 Hz, 4H), 2.63–2.49 (m, 2H). MS: *m/z* 214 (M^+^).

##### 3,4-bis(allyloxy)benzaldehyde (18)

Red oil. ^1^H NMR (300 MHz, CDCl_3_) δ 9.83 (s, 1H), 7.46–7.39 (m, 2H), 6.98 (d, *J* = 8.4 Hz, 1H), 6.17–6.00 (m, 2H), 5.46 (d, *J* = 18.7 Hz, 2H), 5.33 (dd, *J* = 11.2, 6.9 Hz, 2H), 4.69 (dd, *J* = 10.1, 5.2 Hz, 4H). MS: *m/z* 218 (M^+^);

##### 3,4-bis(isopentyloxy)benzaldehyde (19)

Brown oil. ^1^H NMR (400 MHz, CDCl_3_) δ 9.83 (s, 1H), 7.33 (d, *J* = 53.8 Hz, 2H), 6.95 (d, *J* = 8.0 Hz, 1H), 4.08 (dd, *J* = 12.7, 6.5 Hz, 4H), 1.84 (dd, *J* = 12.9, 6.4 Hz, 2H), 1.73 (dd, *J* = 13.3, 6.6 Hz, 4H), 0.97 (d, *J* = 6.5 Hz, 12H). ^13^C NMR (101 MHz, CDCl_3_) δ 191.33, 154.59, 149.52, 130.01, 126.79, 111.84, 110.84, 67.64, 37.91, 37.78, 25.33, 22.75. MS: *m/z* 278 (M^+^); HRMS calcd for C_17_H_26_O_3_: 278.1876 (M^+^), found: 278.1873.

##### 3,4-dipropoxybenzaldehyde (20)

Brown oil. ^1^H NMR (300 MHz, CDCl_3_) δ 9.82 (s, 1H), 7.45–7.38 (m, 2H), 6.96 (d, *J* = 7.9 Hz, 1H), 4.05 (dd, *J* = 14.0, 6.9 Hz, 4H), 1.98–1.81 (m, 4H), 1.07 (dd, *J* = 11.1, 6.8 Hz, 6H). MS: *m/z* 222 (M^+^).

##### 3,4-bis(prop-2-yn-1-yloxy)benzoic acid (21)

White solid. Mp 157–159 °C; ^1^H NMR (400 MHz, CDCl_3_) δ 7.83–7.71 (m, 2H), 7.11 (d, *J* = 8.5 Hz, 1H), 4.83 (dd, *J* = 9.9, 2.4 Hz, 4H), 2.55 (q, *J* = 2.4 Hz, 2H). MS: *m/z* 230 (M^+^).

##### 3,4-bis(isopentyloxy)benzoic acid (22)

White solid. Yield, %. Mp 137–139 °C; ^1^H NMR (300 MHz, CDCl_3_) δ 7.72 (d, *J* = 8.4 Hz, 1H), 7.59 (s, 1H), 6.90 (d, *J* = 8.5 Hz, 1H), 4.10 (q, *J* = 6.3 Hz, 4H), 1.88 (dt, *J* = 13.5, 6.7 Hz, 2H), 1.81–1.69 (m, 4H), 1.00 (d, *J* = 6.5 Hz, 12H). MS: *m/z* 294 (M^+^).

##### 3,4-dipropoxybenzoic acid (23)

White solid. Mp 133–135 °C; ^1^H NMR (300 MHz, CDCl_3_) δ 7.71 (d, *J* = 9.9 Hz, 1H), 7.58 (s, 1H), 6.90 (d, *J* = 8.4 Hz, 1H), 4.04 (dd, *J* = 11.2, 6.6 Hz, 4H), 1.96–1.81 (m, 4H), 1.08 (t, *J* = 7.4 Hz, 6H). MS: *m/z* 238 (M^+^).

##### (*E*)-methyl 3-(3-hydroxy-4-(prop-2-yn-1-yloxy)phenyl)acrylate (24)

White solid. Mp 103–105 °C; ^1^H NMR (400 MHz, CDCl_3_) δ 7.59 (d, *J* = 15.9 Hz, 1H), 7.15 (d, *J* = 2.1 Hz, 1H), 7.03 (dd, *J* = 8.5, 1.9 Hz, 1H), 6.97 (d, *J* = 8.4 Hz, 1H), 6.30 (d, *J* = 15.9 Hz, 1H), 5.67 (s, 1H), 4.79 (d, *J* = 2.4 Hz, 2H), 3.79 (s, 3H), 2.58 (t, *J* = 2.4 Hz, 1H). ^13^C NMR (101 MHz, CDCl_3_) δ 167.75, 146.53, 146.35, 144.56, 129.26, 123.99, 121.59, 116.61, 113.88, 112.59, 111.70, 57.03, 51.78. MS: *m/z* 232 (M^+^); HRMS calcd for C_13_H_12_O_4_: 232.0730 (M^+^), found: 232.0732.

##### (*E*)-methyl 3-(3,4-bis(prop-2-yn-1-yloxy)phenyl)acrylate (25)

White solid. Mp 96–98 °C; ^1^H NMR (400 MHz, CDCl_3_) δ 7.62 (d, *J* = 15.9 Hz, 1H), 7.25–7.11 (m, 2H), 7.04 (d, *J* = 8.4 Hz, 1H), 6.31 (d, *J* = 15.9 Hz, 1H), 4.77 (dd, *J* = 2.4, 1.2 Hz, 4H), 3.79 (s, 3H), 2.57–2.48 (m, 2H). ^13^C NMR (101 MHz, CDCl_3_) δ 167.59, 149.54, 147.63, 144.37, 128.62, 123.25, 116.47, 114.45, 113.93, 78.23, 78.11, 76.44, 76.40, 57.10, 56.85, 51.74. MS: *m/z* 270 (M^+^); HRMS calcd for C_16_H_14_O_4_: 270.0887 (M^+^), found: 270.0888.

#### 3.2.2. General Procedure 2: Synthesis of Compounds **6**, **14**–**15**

A mixture of di-substituted 3,4-dihydroxybenzoic acid methyl ester or 3,4-dihydroxybenzaldehyde with 10% Pd/C in 10% K_2_CO_3_–MeOH was stirred at room temperature for an appropriate time. After the catalyst was filtered, the filtrate was concentrated *in vacuo*. The mixture was extracted with AcoEt. The organic extracts were washed with brine and dried. The solvent was evaporated under reduced pressure to give a residue, which was purified by column-chromatography on silica gel.

##### methyl 3-(allyloxy)-4-hydroxybenzoate (6)

White solid. Mp 46–48 °C; ^1^H NMR (300 MHz, CDCl_3_) δ 7.64 (d, *J* = 10.3 Hz, 1H), 7.56 (s, 1H), 6.96 (d, *J* = 3.9 Hz, 1H), 6.15–6.04 (m, 1H), 6.03 (s, 1H), 5.40 (dd, *J* = 26.7, 13.7 Hz, 2H), 4.68 (d, *J* = 5.5 Hz, 2H), 3.89 (s, 3H). MS: *m/z* 208 (M^+^).

##### 4-hydroxy-3-((3-methylbut-2-en-1-yl)oxy)benzaldehyde (14)

Brown oil. ^1^H NMR (400 MHz, CDCl_3_) δ 9.79 (s, 1H), 7.41 (s, 1H), 7.38 (s, 1H), 7.02 (d, *J* = 7.9 Hz, 1H), 6.53 (s, 1H), 5.46 (t, *J* = 6.3 Hz, 1H), 4.62 (d, *J* = 6.8 Hz, 2H), 1.78 (s, 3H), 1.74 (s, 3H). ^13^C NMR (101 MHz, CDCl_3_) δ 191.02, 151.79, 146.44, 139.68, 129.52, 127.56, 118.24, 114.29, 109.75, 65.77, 25.38, 18.30. MS: *m/z* 206 (M^+^); HRMS calcd for C_12_H_14_O_3_: 206.09387 (M^+^), found: 206.0938.

##### 3-(allyloxy)-4-hydroxybenzaldehyde (15)

Pale white solid. Mp 113–115 °C; ^1^H NMR (300 MHz, CDCl_3_) δ 9.80 (s, 1H), 7.42 (s, 2H), 7.06 (d, *J* = 14.5 Hz, 1H), 6.22 (s, 1H), 6.18–5.98 (m, 1H), 5.41 (dd, *J* = 21.1, 14.9 Hz, 2H), 4.70 (d, *J* = 9.2 Hz, 2H). MS: *m/z* 178 (M^+^).

##### (*E*)-methyl 3-(3,4-dihydroxyphenyl)acrylate (26)

Pale white solid. Mp 164–166 °C; ^1^H NMR (400 MHz, CDCl_3_) δ 7.58 (d, *J* = 22.4 Hz, 1H), 7.08 (d, *J* = 2.0 Hz, 1H), 7.01 (dd, *J* = 8.2, 2.0 Hz, 1H), 6.87 (d, *J* = 8.2 Hz, 1H), 6.27 (d, *J* = 15.9 Hz, 1H), 5.58 (s, 2H), 3.79 (s, 3H). MS: *m/z* 194 (M^+^).

##### methyl 3-(isopentyloxy)-4-methoxybenzoate (27)

Pale yellow oil. ^1^H NMR (400 MHz, CDCl_3_) δ 7.63 (dd, *J* = 8.4, 2.0 Hz, 1H), 7.53 (d, *J* = 2.0 Hz, 1H), 6.85 (d, *J* = 8.5 Hz, 1H), 4.06 (t, *J* = 6.9 Hz, 2H), 3.88 (s, 3H), 3.86 (s, 3H), 1.83 (dt, *J* = 13.0, 6.5 Hz, 1H), 1.74 (q, *J* = 6.9 Hz, 2H), 0.96 (d, *J* = 6.5 Hz, 6H). ^13^C NMR (101 MHz, CDCl_3_) δ 167.02, 153.41, 148.21, 123.32, 122.69, 113.59, 110.49, 67.51, 56.02, 51.98, 37.96, 25.14, 22.67. MS: *m/z* 252 (M^+^); HRMS calcd for C_14_H_20_O_4_: 252.1356 (M^+^), found: 252.1354.

##### 3-methylbut-2-en-1-yl 4-methoxy-3-((3-methylbut-2-en-1-yl)oxy)benzoate (28)

Colorless oil. ^1^H NMR (400 MHz, CDCl_3_) δ 7.59 (d, *J* = 42.4 Hz, 2H), 6.83 (s, 1H), 5.45 (s, 2H), 4.68 (d, *J* = 61.2 Hz, 4H), 3.88 (s, 3H), 1.75 (s, 12H). ^13^C NMR (101 MHz, CDCl_3_) δ 166.61, 153.33, 147.82, 138.86, 138.22, 123.53, 122.87, 119.56, 118.96, 115.70, 113.68, 110.27, 65.97, 61.78, 55.73, 25.90, 18.27, 18.14. MS: *m/z* 304 (M^+^); HRMS calcd for C_18_H_24_O_4_: 304.1669 (M^+^), found: 304.1667.

##### 3-methylbut-2-en-1-yl 3-hydroxy-4-methoxybenzoate (29)

White solid. Mp 154–156 °C; ^1^H NMR (400 MHz, CDCl_3_) δ 7.59 (d, *J* = 6.0 Hz, 2H), 6.82 (d, *J* = 9.0 Hz, 1H), 5.96 (s, 1H), 5.43 (t, *J* = 7.8 Hz, 1H), 4.76 (d, *J* = 7.1 Hz, 2H), 3.88 (s, 3H), 1.74 (d, *J* = 8.1 Hz, 6H). ^13^C NMR (101 MHz, CDCl_3_) δ 166.37, 150.57, 145.25, 138.83, 123.68, 122.64, 118.75, 115.78, 109.93, 61.78, 55.91, 25.58, 18.08. MS: *m/z* 236 (M^+^); HRMS calcd for C_13_H_16_O_4_: 236.1043 (M^+^), found: 236.1042.

#### 3.2.3. Synthesis of 3,4-bis(prop-2-yn-1-yloxy)benzamide (**30**)

The mixture of 3,4-dihydroxybenzoic acid methyl ester and propargyl bromide were dissolved in acetone and 5 equiv K_2_CO_3_ were added. Through general procedure 1, di-substituted compound was obtained. Then it was dissolved in MeOH, 5 equiv of the amine were added and the mixture was stirred at 40 °C for 24 h. The mixture were poured into water and extracted with AcoEt. Removing the water of the aqueous layer and the target compound was obtained as white solid. Mp 145–147 °C; ^1^H NMR (400 MHz, DMSO) δ 7.83 (s, 1H), 7.60–7.49 (m, 2H), 7.22 (s, 1H), 7.10 (d, *J* = 8.5 Hz, 1H), 4.85 (dd, *J* = 14.6, 2.4 Hz, 4H), 3.57 (dt, *J* = 7.6, 2.4 Hz, 2H). ^13^C NMR (101 MHz, DMSO) δ 167.14, 149.27, 146.16, 127.43, 121.25, 113.57, 113.04, 79.03, 78.88, 78.56, 78.44, 56.09, 56.00. MS: *m/z* 230 (M^+^ + H); HRMS calcd for C_13_H_11_NO_3_: 229.0733 (M^+^), found: 229.0732.

##### methyl 3-hydroxy-4-methoxybenzoate (31)

White solid. Mp 65–67 °C; ^1^H NMR (400 MHz, CDCl_3_) δ 7.64–7.57 (m, 2H), 6.87 (d, *J* = 8.4 Hz, 1H), 5.62 (s, 1H), 3.95 (s, 3H), 3.88 (s, 3H). MS: *m/z* 182 (M^+^).

##### (*E*)-methyl 3-(4-hydroxy-3-((3-methylbut-2-en-1-yl)oxy)phenyl)acrylate (32)

Yellow oil. ^1^H NMR (400 MHz, CDCl_3_) δ 7.61 (d, *J* = 15.8 Hz, 1H), 7.06 (d, *J* = 9.6 Hz, 2H), 6.91 (d, *J* = 8.0 Hz, 1H), 6.27 (d, *J* = 15.9 Hz, 1H), 5.96 (s, 1H), 5.48 (s, 1H), 4.61 (s, 2H), 3.79 (s, 3H), 1.79 (d, *J* = 19.9 Hz, 6H). ^13^C NMR (101 MHz, CDCl_3_) δ 167.90, 148.51, 146.09, 145.22, 139.49, 126.93, 123.01, 118.85, 115.21, 114.86, 110.84, 66.03, 51.71, 25.85, 18.39. MS: *m/z* 262 (M^+^); HRMS calcd for C_15_H_18_O_4_: 262.1200 (M^+^), found: 262.1199.

### 3.3. Virus and Cell Culture

The pandemic influenza H1N1 2009 virus Influenza A strains A/GuangdongSB/01/2009 (H1N1), A/Guangdong/03/2009 (H1N1) and A/Guangdong/05/2009 (H1N1) were kindly provided by Dr. Li at the Sun Yat-sen University Zhongshan School of Medicine (Guangzhou, Guangdong, China.) who isolated it during the human swine flu outbreak of 2009. Madin-Darby canine kidney (MDCK) cells were cultured in DMEM (Invitrogen, Carlsbad, CA, USA) supplemented with 10% fetal bovine serum (FBS) (Hyclone, Logan, UT, USA), 2 mM l-glutamine, 100 μg/mL streptomycin and 100 units/mL penicillin (Invitrogen, Carlsbad, CA, USA). The cultures were maintained at 37 °C in a humidified atmosphere of 5% CO_2_. All viruses were amplified and titrated in MDCK cells and stored at −80 °C until use. Confluent MDCK monolayers in 6-well plates were washed three times with PBS and incubated with viruses at a multiplicity of infection (MOI) of 0.001 in DMEM for 2 h at 37 °C. After viral adsorption, media was removed, cells were washed three times with PBS and incubated with post-adsorption medium [DMEM, supplemented with 1% fetal bovine serum (FBS) (Hyclone, Logan, UT, USA), 2 mM l-glutamine, 100 μg/mL streptomycin and 100 units/mL penicillin (Invitrogen, Carlsbad, CA, USA), 25 mM HEPES buffer (pH 7.2), 0.1 mM non-essential amino acids, 1.0 mM sodium pyruvate, 2 mg/mL l-1-(tosylamido-2-phenyl) ethyl chloromethyl ketone (TPCK)-treated trypsin (Invitrogen, Carlsbad, CA, USA)].

### 3.4. Neuraminidase Activity Inhibition Assay

Influenza virus neuraminidase (NA) sensitivity to the test compounds was evaluated by using an NA activity inhibition assay, based on the method of Maki Kiso *et al.* [11]. Briefly, 4-Methylumbelliferyl-*N*-acetylneuraminic acid (4-MUNANA, Sigma-Aldrich, St. Louis, MO, USA), at a final concentration of 0.15 mM, was used as the fluorescent substrate. The virus solution [(A/Guangdong/03/2009 (H1N1), ranging from 800 to 1200 fluorescence units of MU-NANA] was prepared in MES assay buffer [32.5 mM MES (pH 6.5), 4 mM CaCl_2_, 0.1% NP-40, 0.3 mg/mL BSA], the test compounds were dissolved in MES assay buffer at various concentrations (1, 10, 20, 40, 50, 80 μM), and Tamiflu^®^ (Roche Laboratories Inc., Basel, Switzerland) was used as a positive control. Diluted virus was mixed with each test compound in 50 μL MES assay buffer, and the mixtures were incubated at 37 °C for 1 h with gentle agitation, then mixed with 50 μL 4-MUNANA buffer and incubated at 37 °C for another 1 h with gentle agitation. The reaction was terminated by adding 100 μL of 0.1 M NaOH in 80% ethanol (pH 10.0). Then the fluorescence was measured at an excitation wavelength of 360 nm and an emission wavelength of 465 nm, using a SpectraMax M5 multi-detection reader (Molecular Devices Corporation, Sunnyvale, CA, USA). Under the experimental conditions, a linear relationship between the enzyme amount and the fluorescence was confirmed. Each NA inhibition assay was done in triplicate. NA activity inhibition was determined using the following formula as described previously [11]: NA activity inhibition (%) = (1 − OD_465_ of treated group/OD_465_ of control group) × 100%. The half maximal inhibitory concentration (IC_50_) was calculated by Bliss software [12] and the data, expressed as means ± SD, were analyzed by SPSS.

### 3.5. Modelling of Enzyme-Substrate Complexes by Molecular Docking

Surflex-Dock was applied to study molecular docking. Crystal structure of NA was retrieved from RCSB Protein Data Bank (PDB entry code: 2ht7) [13]. This is a particular structure with oseltamivir. The protein structure was utilized in subsequent docking experiments without energy minimization. All ligands and water molecules have been removed at first and the polar hydrogen atoms were added. Automatic docking was employed. Other parameters were established by default in software. All molecules were minimized using default parameter.

## 4. Conclusions

This study provided a new kind of non-nitrogenous small molecule NA inhibitor, some examples of which had stronger activities than the reported benzoic acid compounds that have high affinity for influenza virus but high IC_50_ values (~mM level) [7]. It also proved that the carboxyl group is not necessary and replacement with an aldehyde group enhanced the activity. The OH group was indispensable to the activity, but the ether chains were not crucial to the activity, regardless of their degree of unsaturation. In addition, these compounds were easily obtained with excellent yields. Further studies aimed at developing an efficient anti-influenza virus candidate based on the SAR analysis and the docking results are underway in our laboratory.
